# Integrated transcriptome and metabolome analysis reveals the regulation of phlorizin synthesis in *Lithocarpus polystachyus* under nitrogen fertilization

**DOI:** 10.1186/s12870-024-05090-9

**Published:** 2024-05-06

**Authors:** Suping Zeng, Longhua Yu, Ping He, Hui Feng, Jia Wang, Huacong Zhang, Yunxia Song, Ren Liu, Yueqiao Li

**Affiliations:** 1 Experimental Center of Subtropical Forestry, Chinese Academy of Forestry, Xinyu, 336600 China; 2 Research Institute of Forestry, Chinese Academy of Forestry, Beijing, 100091 China; 3 Xinyu University, School of Public Health and Health, Xinyu, 338004 China

**Keywords:** Nitrogen fertilization, Transcriptomics, Metabolomics, *Lithocarpus Polystachyus*, Phlorizin

## Abstract

**Background:**

Nitrogen (N) is essential for plant growth and development. In *Lithocarpus polystachyus* Rehd., a species known for its medicinal and food value, phlorizin is the major bioactive compound with pharmacological activity. Research has revealed a positive correlation between plant nitrogen (N) content and phlorizin synthesis in this species. However, no study has analyzed the effect of N fertilization on phlorizin content and elucidated the molecular mechanisms underlying phlorizin synthesis in *L. polystachyus*.

**Results:**

A comparison of the *L. polystachyus* plants grown without (0 mg/plant) and with N fertilization (25, 75, 125, 175, 225, and 275 mg/plant) revealed that 75 mg N/plant fertilization resulted in the greatest seedling height, ground diameter, crown width, and total phlorizin content. Subsequent analysis of the leaves using ultra-performance liquid chromatography-tandem mass spectrometry (UPLC-MS/MS) detected 150 metabolites, including 42 flavonoids, that were differentially accumulated between the plants grown without and with 75 mg/plant N fertilization. Transcriptomic analysis of the *L. polystachyus* plants via RNA sequencing revealed 162 genes involved in flavonoid biosynthesis, among which 53 significantly differed between the N-treated and untreated plants. Fertilization (75 mg N/plant) specifically upregulated the expression of the genes phenylalanine ammonia-lyase (*PAL*), 4-coumarate-CoA ligase (*4CL*), and phlorizin synthase (*PGT1*) but downregulated the expression of trans-cinnamate 4-monooxygenase (*C4H*), shikimate O-hydroxycinnamoyltransferase (*HCT*), and chalcone isomerase (*CHI*), which are related to phlorizin synthesis. Finally, an integrated analysis of the transcriptome and metabolome revealed that the increase in phlorizin after N fertilization was consistent with the upregulation of phlorizin biosynthetic genes. Quantitative real-time PCR (qRT‒PCR) was used to validate the RNA sequencing data. Thus, our results indicated that N fertilization increased phlorizin metabolism in *L. polystachyus* by regulating the expression levels of the *PAL*, *PGT1*, 5-O-(4-coumaroyl)-D-quinate 3’-monooxygenase (*C3’H*), *C4H*, and *HCT* genes.

**Conclusions:**

Our results demonstrated that the addition of 75 mg/plant N to *L. polystachyus* significantly promoted the accumulation of flavonoids, including phlorizin, and the expression of flavonoid synthesis-related genes. Under these conditions, the genes *PAL*, *4CL*, and *PGT1* were positively correlated with phlorizin accumulation, while *C4H*, *CHI*, and *HCT* were negatively correlated with phlorizin accumulation. Therefore, we speculate that *PAL*, *4CL*, and *PGT1* participate in the phlorizin pathway under an optimal N environment, regulating phlorizin biosynthesis. These findings provide a basis for improving plant bioactive constituents and serve as a reference for further pharmacological studies.

**Supplementary Information:**

The online version contains supplementary material available at 10.1186/s12870-024-05090-9.

## Background

*Lithocarpus polystachyus* (Wall.) Rehd., also known as multispike tea or sweet tea, belongs to the Fagaceae family [1]. Its leaves are rich in flavonoids and polyphenols but low in caffeine and reducing sugars. The primary active compounds in *L. polystachyus* leaves are flavonoids [2], of which phloretin, phlorizin and quercetin are the major ones [3]. Phlorizin is known for its various pharmacological activities, such as hypoglycemic, hypolipidemic, antioxidant, anticancer, anti-osteoporosis, anti-hepatic fibrosis, and anti-ischemic effects [4, 5]. In addition, its oxidized product is used as a natural dye in the food processing industry [6]. Thus, *L. polystachyus* is highly valued as a beverage and medicine [7, 8] and has unlimited prospects in drug research and development and functional food development [9]. Phlorizin was first isolated from a domesticated apple (*Malus pumila* Mill.) in 1835 and was later detected in high amounts in *Malus* species and in small quantities in *Litchi chinensis* Sonn., *Pyrus betulifolia* Bunge, and *Cynomorium songaricum* Rupr. Recently, researchers noticed high levels of phlorizin in *L. polystachyus* leaves [3, 10]. Improving phlorizin synthesis and accumulation and obtaining authentic raw materials will help further enhance the application value of *L. polystachyus*.

Research on *L. polystachyus* has focused primarily on chemical component analysis [11], extraction [12], functional verification [13], artificial cultivation [9], chloroplast genome sequencing, and functional gene cloning [1]. The environmental factors influencing the active ingredients of *L. polystachyus* and the differences in the active ingredients under various meteorological and soil conditions have also been analyzed [9, 14, 15]. For example, Yang et al. [15] reported that latitude, annual average temperature, extreme minimum temperature, soil pH, soil organic matter, and soil nitrogen (N) influence the composition of bioactive components. He et al. [16] established a significant positive correlation between phlorizin and total nitrogen (N) in *L. polystachyus* leaves, while Li et al. [17] correlated the N fertilizer applied with the leaf N content. However, the impact of N fertilization on the active components of *L. polystachyus* remains unknown. Understanding the correlation between N application and phlorizin content will aid in planning precision N fertilization for *L. polystachyus* in artificial forests.

Nitrogen (N) is an essential nutrient involved in several plant biological processes. The N applied to the soil regulates photosynthesis and influences primary and secondary metabolite biosynthesis [18]. Several studies have interpreted the effects of soil N levels on flavonoids through transcriptomics, metabolomics, or multiomics. Through transcriptomics, Ye et al. [19] showed that light intensity and spectral composition regulate flavonoid biosynthesis in *Camellia sinensis*; here, high light intensity downregulated key genes of the flavonoid pathway [*PAL*, chalcone synthase (*CHS*), and flavanone 3-hydroxylase (*F3H)*]. Wang et al. [20] showed that an increase in pure N applied to soil from 22.5 kg/hm^2^ to 67.5 kg/hm^2^ enhanced PAL activity and flavonoid content in the upper leaves of flue-cured tobacco (*Nicotiana tabacum* L.). Blume et al. [21] correlated PAL activity with increased flavonoids and phenolics in *Labisia pumila* (Blume) Fern. under a low N gradient (0 ∼ 90 kg N/ha). However, in plants, the synthesis of phlorizin is different from that of other dihydrochalcones, and the influence of N on phlorizin synthesis remains elusive. The synthesis of phlorizin in apples has been reported, and here, phlorizin is synthesized via two pathways (A and B) from the precursors malonyl CoA and p-coumaroyl CoA [22]. In the first pathway, chalcone synthetase (CHS) first catalyzes the conversion of malonic acid, monophthalide CoA, and p-coumaroyl CoA to naringenin chalcone, which is further converted into other flavonoids. In the second pathway, p-coumaroyl CoA is converted to 4-hydroxycinnamoyl CoA (4-hydrocinnamoyl CoA) through reduced coenzyme II (NADPH). Furthermore, under the action of CHS, malonic acid, monoacyl CoA, and 4-hydrocinnamoyl CoA produce phloretin, which is glycosylated to phlorizin. A recent study on the effect of N fertilizer in apple demonstrated a decrease in the expression of flavonoid biosynthesis-associated proteins (PGT1, F3H, and ANR) and the synthesis of phloretin but not phlorizin [23]. However, the effects of phlorizin synthesis and N fertilization on the transcriptional regulation of phlorizin metabolism in *L. polystachyus* remain unclear.

Among the various metabolomics and transcriptomics approaches, ultra-performance liquid chromatography-tandem mass spectrometry (UPLC-MS/MS)-based on widely targeted metabolomics has been widely used to analyze and identify metabolites in plants such as rice (*Oryza sativa* L.) [24], tomato (*Solanum lycopersicum* L.) [25, 26], and maize (*Zea mays* L.) [27]. This rapid and highly accurate method adopts a multiple reaction monitoring (MRM) mode to qualitatively and quantitatively analyze metabolites based on the self-established Metware database (MWDB) [24, 28]. In addition, integrating transcriptomics with metabolomics has proven useful for exploring the mechanisms underlying the biosynthesis of key metabolites [29–31]. Therefore, we assume that this integrated approach will aid in the analysis of the flavonoid biosynthetic pathway in *L. polystachyus*, especially in response to N fertilization.

Although high N increases plant yield and quality, excessive N application affects the C–N balance and negatively influences bioactive components, such as flavonoids. Therefore, we investigated the effect of N application on the growth and bioactivity of *L. polystachyus* via a pot experiment. We assessed the growth status and leaf phlorizin content of *L. polystachyus* under different N fertilization levels and determined the molecular mechanism underlying the N application-associated regulation of bioactive synthesis in the fertilized sample with the best growth and quality. Studies have reported the influence of N fertilization on secondary metabolites and the positive correlation between N amount and phlorizin. Therefore, we hypothesized that moderate N application could increase the phlorizin content in *L. polystachyus* leaves, while excessive N could cause toxic effects. Specifically, an optimal level of N fertilization could affect the synthesis of phlorizin by regulating the expression of key biosynthetic genes. These findings will help elucidate the mechanism of phlorizin biosynthesis in *L. polystachyus* under optimal N fertilization. This study will also provide a scientific basis for applying N fertilization to improve the bioactive content and quality of *L. polystachyus* and similar economically important crops.

## Results

### Changes in the physiological parameters and bioactive components of *L. Polystachyus* in response to N fertilization

**Fig. 1:**
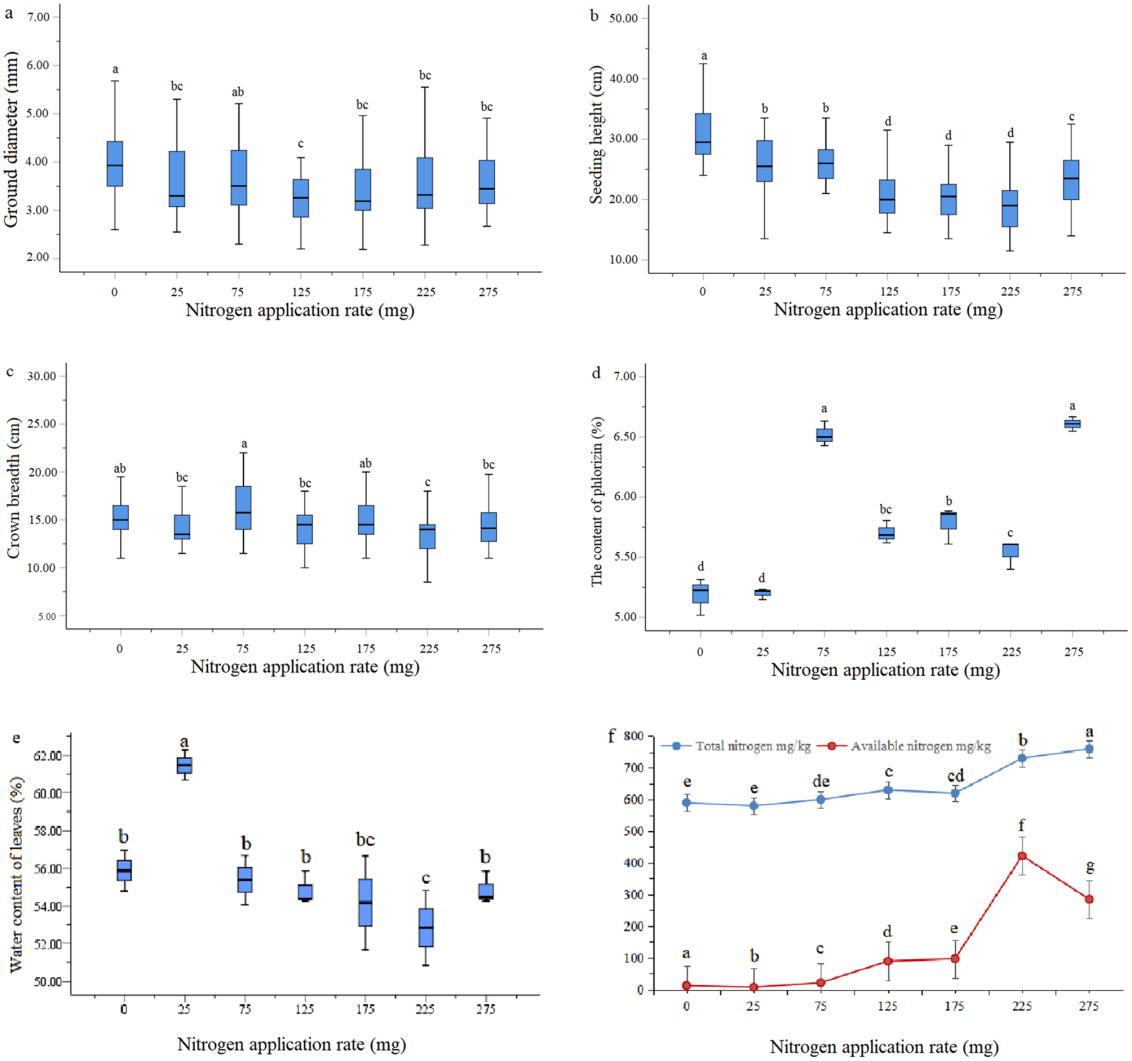
Changes in the physiological parameters and bioactive components of *L. polystachyus* in response to N fertilization. (**a**) Ground diameter, (**b**) seedling height, (**c**) crown width, (**d**) leaf phlorizin content, (**e**) leaf water content, and (**f**) soil total nitrogen and available nitrogen of *L. polystachyus* under N fertilization ranging from 0 to 275 mg/plant. The data shown are the means ± standard errors (SEs, *n* = 42). Different lowercase letters above the bars indicate significant differences at the 0.05 level

In this study, we first analyzed the effects of N fertilization at various concentrations (0, 25, 75, 125, 175, 225, and 275 mg/plant) on the physiological parameters and effective components of *L. polystachyus* (Fig. 1). With increasing N levels, *L. polystachyus* exhibited a decrease in ground diameter (Fig. 1a). Compared with no N fertilization, N fertilization at all levels except for 75 mg N/plant significantly lowered the ground diameter (*P* < 0.05). An increase in the N level also reduced the seedling height (Fig. 1b). Moreover, the crown width first increased (0 ∼ 75 mg N/plant) and then decreased (125 ∼ 275 mg N/plant) as the N fertilization rate increased (Fig. 1c); crown width was greatest (16.25 ± 3.01 cm) for *L. polystachyus* plants under 75 mg N/plant fertilization. This specific level of N fertilization also resulted in the maximum ground diameter and seedling height.

Further analysis of the bioactive components in *L. polystachyus* leaves revealed that the content of phlorizin increased significantly after N fertilization (Fig. 1d). The application of N at 75 and 275 mg/plant resulted in a substantially greater phlorizin content than the other N application levels did. However, 275 mg/plant N fertilization had a toxic effect on leaf growth. Thus, based on these initial experiments, we concluded that a 75 mg/plant N application rate had the greatest effect on the bioactive components of *L. polystachyus* leaves. The water content of leaves was the highest at 25 mg/plant N fertilization, while little difference was detected for the other N levels (Fig. 1e). Our results also revealed that the total N and available N contents in the soil increased with increasing N application rates (0 ∼ 275 mg/plant, Fig. 1f). A significantly greater content of total N was found at 225 ∼ 275 mg N/plant fertilization. Moreover, significant differences were detected in soil available N among the N levels. Thus, based on the physiological parameter and bioactive component data, plants treated with 0 and 75 mg/plant N were selected for subsequent assays, such as metabolite profiling and transcriptome analysis, to elucidate the impact of N fertilization on phlorizin synthesis in *L. polystachyus* leaves.

### Qualitative and quantitative analyses of metabolites in *L. Polystachyus* leaves

**Fig. 2:**
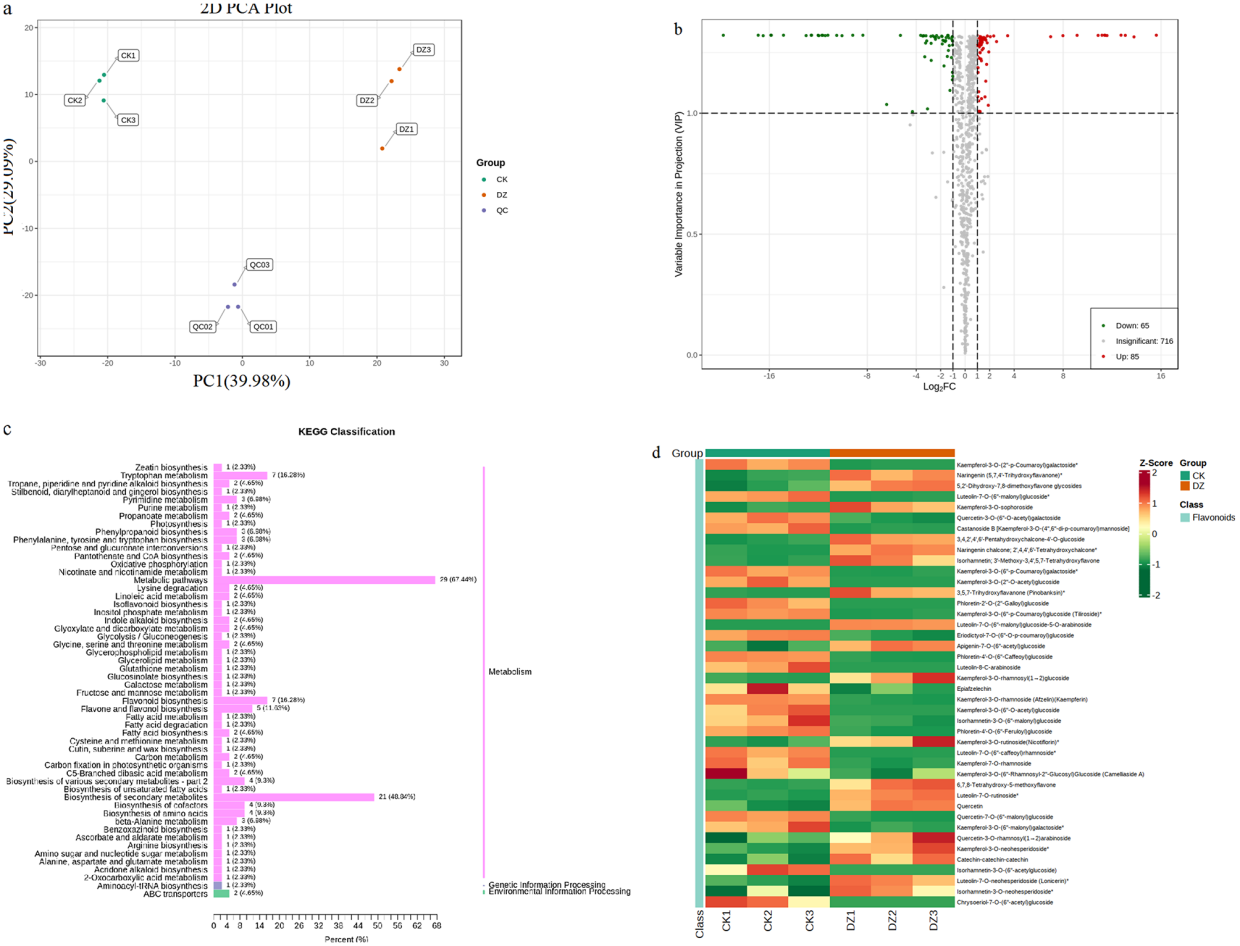
Differentially abundant metabolites in the leaves of *L. polystachyus* under nitrogen fertilization. **a** Principal component analysis (PCA), **b** volcano plot, and **c** KEGG classification of the differentially abundant metabolites identified between the leaves of control (CK, 0 mg N/plant) and treated (DZ, 75 mg N/plant) plants. In b, each point represents a metabolite; the horizontal and vertical coordinates represent the log of the quantitative difference in a metabolite and the VIP values, respectively. Red and green points represent the upregulated and downregulated metabolites, respectively, while gray points represent the metabolites with no significant difference. The values and percentages indicate the number and proportion of each metabolite. **d** Clustering heatmap showing the contents of the significantly different flavonoids. Columns and rows in the heatmap represent samples and metabolites, respectively. The colors indicate the differences in the content of metabolites; red represents high metabolite content, and green represents low metabolite content

Metabolite profiling of the leaves of *L. polystachyus* under two N fertilization levels (0 and 75 mg N/plant) revealed 867 metabolites, including 164 flavonoids, 152 phenolic acids, 137 lipids, 92 others, 6 tannins, 87 amino acids and derivatives, 78 organic acids, 59 nucleotides and derivatives, 57 alkaloids, 27 lignans and coumarins, 7 terpenoids, and 1 steroid (Table S2). Overall, the accumulation of flavonoids increased in response to N fertilization (Fig. 2). Among the 164 flavonoids, 18 were chalcones, of which glucoside (13) and phlorizin (Table S2) were the most abundant. Furthermore, PCA was performed to detect the overall differences in the metabolites between the two samples. The first (PCA1, 39.98%) and second (PCA2, 29.09%) axes explained 69.07% of the total variance between the samples. In addition, the PCA scatter plot revealed a clear separation of the control (0 mg/plant N rate; CK) and fertilized (75 mg/plant N rate; DZ) samples, indicating a significant change in metabolites after N fertilization (Fig. 2a). PCA also revealed less variation between the biological replicates of the same group. Further analysis based on OPLS-DA identified 150 metabolites that were significantly different between the plants grown without and those grown with N fertilization. Among them, 85 metabolites were upregulated, and 65 were downregulated (Fig. 2b). Flavonoids, phenolic acids, and lignans decreased after N application, while lipids, amino acids and derivatives, and organic acids increased (Table S3). Specifically, flavonoids, including phlorizin, were upregulated in the leaves of *L. polystachyus* after N application.

KEGG enrichment analysis revealed that the 150 significantly different metabolites were enriched in 54 metabolic pathways (Fig. 2c), including tryptophan metabolism, flavonoid biosynthesis, flavone and flavonol biosynthesis, cofactor biosynthesis, amino acid biosynthesis, and beta-alanine metabolism. Seven metabolites were enriched in the tryptophan metabolism and flavonoid biosynthesis pathways, five were enriched in the flavone and flavonoid biosynthesis pathways, four were enriched in the biosynthesis of cofactors and biosynthesis of amino acids, and three were enriched in beta-alanine metabolism. The clustering heatmap showed that the metabolites that differentially accumulated after N fertilization were mainly flavonoids. A total of 42 significantly different flavonoids were detected in the plants with and without N fertilization (Fig. 2d). Among these genes, 19 were upregulated after N fertilization, including one flavanol (catechin), one flavanone (naringenin), one flavanonol (pinobanksin), two chalcones, six flavones, and eight flavonols, while 23, including 1 flavanol, 1 flavanone, 3 chalcones (phloretin), 4 flavones, and 14 flavonols, were downregulated.

### Transcriptome of *L. Polystachyus* under N fertilization

**Fig. 3:**
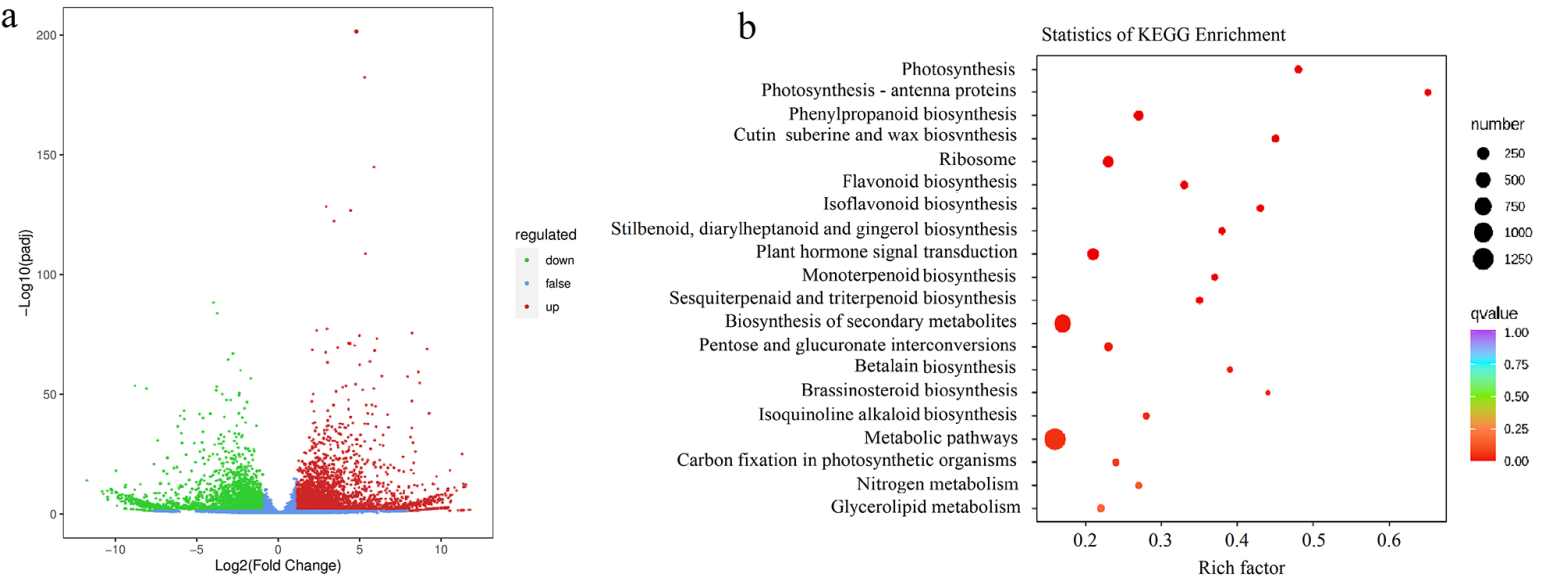
Differential expression of genes in the leaves of *L. polystachyus* under N fertilization. **a** Volcano plot and **b** KEGG enrichment analysis of differentially expressed genes. In a, the abscissa and ordinate represent the log_2_-fold change and − log_10_ false discovery rate, respectively. Red and green dots indicate upregulated and downregulated genes, respectively; blue dots indicate genes whose expression did not significantly change after N application

**Fig. 4:**
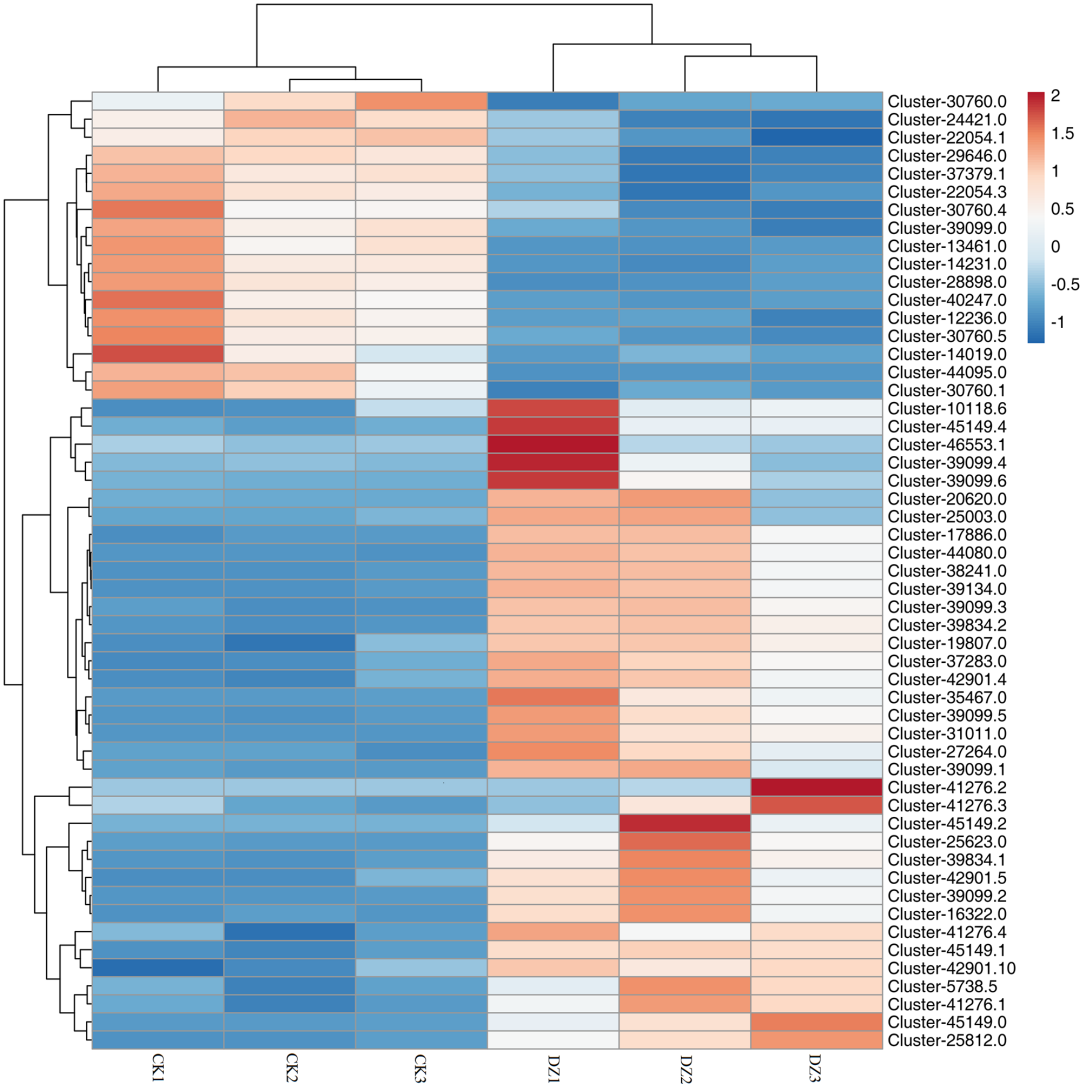
Clustered heatmap showing the expression of genes associated with flavonoid biosynthesis in the leaves of *L. polystachyus* under N fertilization. Columns and rows represent samples and genes, respectively. Gene expression levels are represented by different colors; red indicates high expression, and blue indicates low expression. ^a^CK: no N fertilization; DZ: 75 mg N/plant

We further sequenced the leaf transcriptome of *L. polystachyus* under two N fertilization levels. Approximately 6.78 G of clean bases, with a Q30 of 93.31%, were obtained (47,216,097 raw reads and 45,213,021 clean reads) from the untreated samples, and 6.70 G of clean bases (46,132,578 raw reads and 44,636,161 clean reads), with a Q30 of 92.69%, were obtained from the samples under N fertilization (Table 1). The differentially expressed genes (DEGs) among these two samples are shown in Fig. 3. A total of 7447 DEGs, including 3430 upregulated and 4017 downregulated DEGs (DESeq2; |log_2_(fold change)| ≥1 and FDR < 0.05), were detected between the *L. polystachyus* plants with and without N fertilization (Fig. 4a).

Furthermore, the DEGs related to flavonoid metabolism were screened using the KEGG database (Fig. 4b). This approach identified 86 DEGs involved in phenylpropane metabolism, such as flavonoid biosynthesis (ko00941), isoflavonoid biosynthesis (ko00943), and flavone and flavonol biosynthesis (ko00944) pathways. Most DEGs identified between the plants without and with N fertilization were associated with flavonoid biosynthesis (53), followed by isoflavonoid biosynthesis (29) and flavone and flavonol biosynthesis (4). In addition, 731 DEGs were enriched in the biosynthesis of secondary metabolites; among these, 171, 141, and 119 DEGs participated in the ribosome, MAPK signaling pathway-plant, and phenylpropanoid biosynthesis pathways, respectively (Fig. 4b; Table S4).

KEGG enrichment analysis revealed that flavonoid biosynthesis was the significantly enriched metabolic pathway related to phlorizin in the CK (control) vs. DZ (treated) comparison; 53 DEGs, including 36 upregulated and 17 downregulated DEGs, enriched this pathway (Table S5). Among these DEGs, those encoding the CHR (Cluster-10118.6), PGT1 (Cluster-39834.1, Cluster-39834.2), and C3’H (Cluster-16322.0) enzymes were upregulated, while those encoding C4H (Cluster-14019.0) and chalcone synthase (CHS, Cluster-24421.0) were downregulated (Fig. 3). These observations thus revealed an increase in some flavonoids and *CHR-*, *PGT1-*, and *C3’H-*encoding genes at a low N fertilization level (75 mg/plant) within 90 days, indicating that *CHR*, *PGT1*, and *C3’H* are critical in regulating flavonoid synthesis under these conditions.

**Table 1:** Summary of sequencing data

Sample	Raw Reads	Clean Reads	Clean Base (G)	Error Rate (%)	Q20 (%)	Q30 (%)	GC Content (%)
T^a^	45,343,104	43,196,672	6.48	0.03	97.76	93.38	44.46
	48,094,716	45,870,902	6.88	0.03	97.70	93.24	44.37
	48,210,472	46,571,488	6.99	0.03	97.73	93.32	44.42
F^a^	44,816,252	43,732,208	6.56	0.03	97.58	92.93	43.90
	45,861,466	44,234,876	6.64	0.03	97.45	92.70	43.85
	47,720,016	45,941,398	6.89	0.03	97.32	92.45	44.24

^a^T represents the control group, and F represents the experimental group

### Integrating analysis of the transcriptome and metabolome associated with flavonoid biosynthesis in the leaves of *L. Polystachyus* under N fertilization

**Fig. 5:**
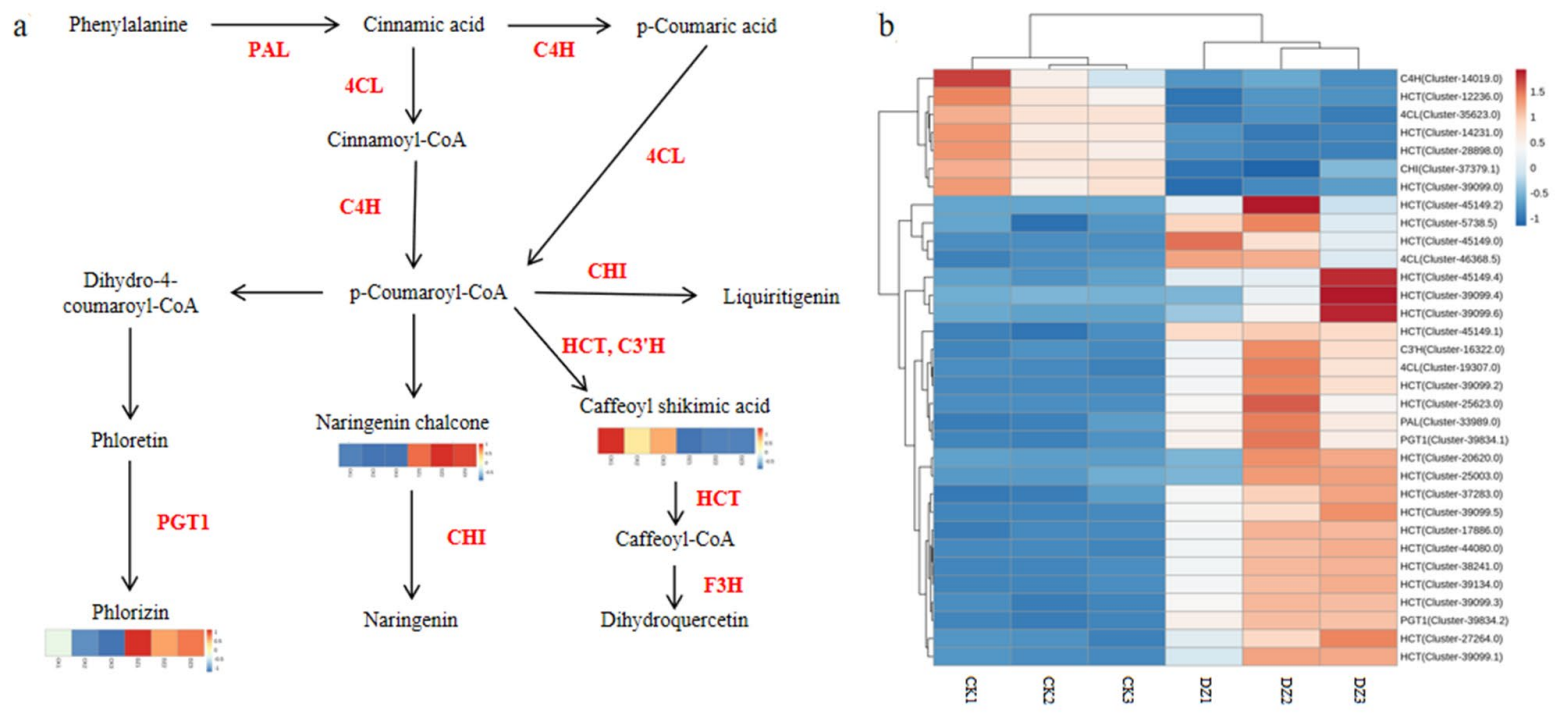
Proposed pathway of phlorizin synthesis in *L. polystachyus* leaves. **(a)** Metabolic pathways associated with phlorizin synthesis. **(b)** The heatmap shows the expression of genes associated with phlorizin synthesis. The KEGG database was used as a reference to construct the metabolic pathway. The metabolic components were mapped to generate the flavonoid metabolic pathway, including phenylpropanoid biosynthesis (ko00940) and flavonoid biosynthesis (ko00941). In the heatmap, red indicates high metabolite content, and blue indicates low metabolite content

Combining the results obtained from transcriptome and metabolome profiling, an integrated map was generated for the metabolites and genes associated with phlorizin biosynthesis (Fig. 5). Initially, we mapped the metabolic components in the leaves of *L. polystachyus* to genes involved in flavonoid metabolism (ko00940 and ko00941). Subsequently, we combined the differentially accumulated metabolites with the DEGs to elucidate the key genes associated with phlorizin biosynthesis under N fertilization (Fig. 5). This integrated approach (Fig. 5a) showed that PAL*-*assisted cinnamic acid synthesis is the first step in the phlorizin biosynthetic pathway. The enzyme C4H catalyzes the formation of cinnamoyl-CoA from p-coumaroyl-CoA, while HCT and C3’H catalyze the formation of caffeoyl shikimic acid mediated by shikimate O-hydroxycinnamoyltransferase and 5-O-(4-coumaroyl)-D-quinate 3’-monooxygenase. Furthermore, PGT1 catalyzes the conversion of phloretin to phloretin-2’-O-glucoside (phlorizin). In this study, unigenes specifically involved in phlorizin synthesis, including one *PAL*, one *C4H*, three *4CL*, one *CHI*, twenty-four *HCT*, one *C3’H*, one *F3H*, and two *PGT1* genes, were screened from the DEGs. In addition, multiple genes encoding the same enzyme were found. Specifically, four *PAL* and five *4CL* genes were identified in *L. polystachyus* under N fertilization. Furthermore, we found that N fertilization upregulated the expression of the *PAL* (Cluster-33989.0), *4CL* (Cluster-46368.5), *PGT1* (Cluster-39834.1, Cluster-39834.2), *C3’H* (Cluster-16322.0), and *F3H* genes of the flavonoid biosynthetic pathway and downregulated the expression of *4CL* (Cluster-35623.0), *C4H* (Cluster-14019.0), *CHI* (Cluster-37379.1), and *HCT* (Cluster-12236.0, Cluster-14231.0, Cluster-28898.0, Cluster-39099.0) genes (Fig. 5b), which could promote the biosynthesis of chalcones, such as phlorizin. Although the expression of *C4H*, a direct regulatory gene in the phlorizin biosynthetic pathway, showed a downward trend, the content of p-coumaroyl-CoA synthesized by C4H showed an increasing trend after N application (Table S2). In addition, the upstream genes *PAL* and *4CL* and the downstream gene *PGT1* were upregulated, indicating that the phlorizin content increased under the comprehensive regulation of upstream and downstream genes. These results showed that *PAL* (Cluster-33989.0), *4CL* (Cluster-46368.5, Cluster-19307.0), *C4H* (Cluster-14019.0), *PGT1* (Cluster-39834.1, Cluster-39834.2), *CHI* (Cluster-37379.1), and *HCT* (Cluster-12236.0, Cluster-14231.0, Cluster-28898.0, Cluster-39099.0) are the primary genes associated with phlorizin synthesis in *L. polystachyus* under N fertilization. Moreover, under N fertilization, the expression levels of *PAL*, *4CL*, and *PGT1* increased, while those of *C4H*, *CHI*, and *HCT* decreased, indicating that N fertilization reduced the conversion of p-coumaroyl-CoA to caffeoylshikimic acid and promoted the formation of phlorizin.

**Fig. 6:**
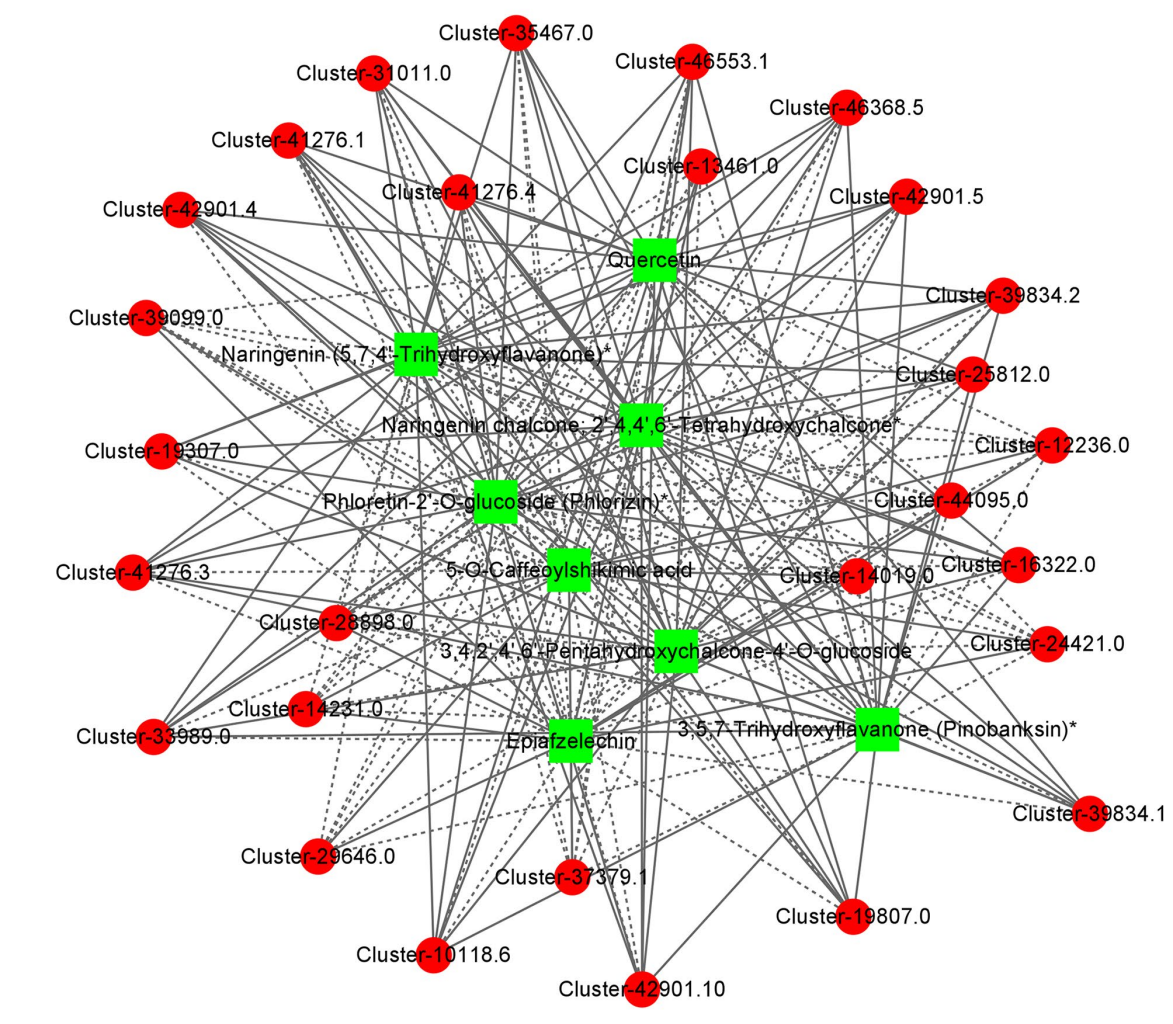
Correlations between the differentially expressed genes (DEGs) and differentially accumulated metabolites of the flavonoid biosynthetic pathway in *L. polystachyus* leaves under N fertilization. Metabolites are shown in green squares, and genes are shown in red circles. The solid lines represent positive correlations, and the dashed lines represent negative correlations

Furthermore, positive correlations were found between the expression levels of most genes and the content of total flavonoids. The network generated using the DEGs and differentially accumulated metabolites associated with flavonoid biosynthesis showed that the genes and metabolites were highly connected (Fig. 6). For example, a *PAL* unigene (Cluster-33989.0) and two *PGT1* unigenes (Cluster-39834.1 and Cluster-39834.2) showed significant correlations with eight metabolites in the flavonoid pathway; among these, six metabolites were positively correlated, and two were negatively correlated. Most DEGs showed significant correlations with phlorizin in the network. Among them, *PAL*, *4CL* (Cluster-19307.0, Cluster-46368.5), and *PGT1* were positively correlated with phlorizin, while *C4H* (Cluster-14019.0), *CHI* (Cluster-37379.1), and *HCT* (Cluster-14231.0, Cluster-28898.0, Cluster-39099.0, Cluster-12236.0) were negatively correlated.

### qRT‒PCR validation of differentially expressed flavonoid biosynthetic genes

**Fig. 7:**
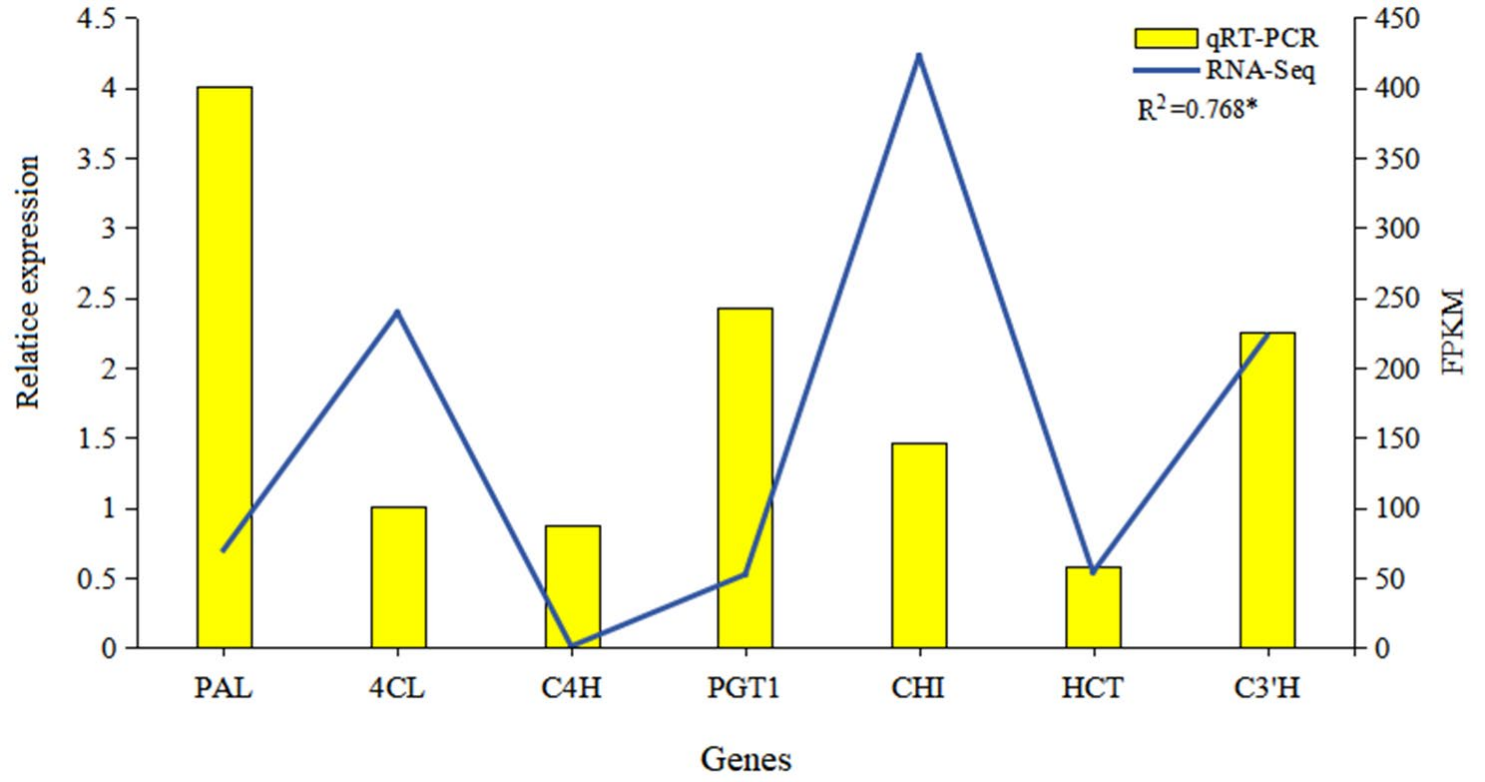
Validation of the expression of key genes associated with flavonoid biosynthesis in the *L. polystachyus* leaf transcriptome. The R^2^ represents the Pearson coefficient between the qRT‒PCR and RNA‒seq results, and asterisks indicate significant differences (*p < 0.05)

To verify the accuracy of the transcriptome sequencing data, qRT‒PCR was used to analyze the expression levels of flavonoid biosynthesis-associated genes (*PAL*, *4CL*, *C4H*, *PGT1*, *CHI*, *HCT*, and *C3’H*) (Fig. 7). The qRT‒PCR results of these genes generally corresponded with the trends of FPKM values obtained by RNA-Seq, except for *CHI*. Furthermore, significant correlations existed between the qRT‒PCR results (2^−ΔΔCt^) and the RNA-Seq results (FPKM), confirming the reliability of the RNA-Seq results.

## Discussion

### Effects of N fertilization on the physiological parameters and active components of *L. Polystachyus*

The effects of exogenous factors, such as N application, on flavonoids have been reported in various species. For example, the total flavonoid content in *Labisia pumila* Blume gradually decreased with an increase in the N application rate from 0 kg/hm^2^ to 270 kg/hm^2^ [32]. However, excessive N inhibits plant growth. Wang et al. [33] showed that an optimal N application level promoted the seedling height and stem biomass of *Catalpa bungei* clones; however, excessive N application inhibited growth. Duan et al. [34] reported that the height, basal diameter, and biomass of *Nitraria tangutorum* Bobr. first increased and then decreased with increasing N level. In this study, N application increased the crown width, ground diameter, and seedling height of *L. polystachyus*, and these traits were greatest at the 75 mg N/plant fertilization rate. These observations were consistent with those of Gao et al. [35], who reported an increase in spring soybean configuration and canopy structure with N fertilization. Similarly, Forsmark et al. [36] reported a significant increase in the needle biomass and fine roots of *Pinus sylvestris* L. under N fertilization. In addition to the plant parameters, the available N content of the soil also increased with increasing fertilizer application level in this study. Thus, the present and earlier studies concluded that N fertilization increases the available N in the soil and increases the amount of N absorbed by plants. This increase in N utilization enhances photosynthetic pigments and reproductive growth, improving growth.

Several researchers have reported the impact of abiotic factors on the active ingredients of *L. polystachyus* [9, 14, 15]. Yang et al. [15] reported that climate (latitude, annual average temperature) and soil physical and chemical properties (pH, organic matter, and N content) influenced the bioactive components of *L. polystachyus*. Our results revealed that a low level of N fertilization (75 mg/plant) had the greatest effect on the bioactive components in *L. polystachyus* leaves. This observation was consistent with the accumulation of flavonoids and total flavonoids detected at the early stage of treatment (50 d and 83 d) in *Nicotiana tabacum* L. under low N fertilization (60 kg/hm^2^). Notably, the upregulation of genes related to flavonoid metabolism resulted in high flavonoid accumulation under N fertilization [37]. In addition, He et al. [16] reported a significant positive correlation between phlorizin and total N content (13.64–17.05 mg/g) in *L. polystachyus* leaves.

Generally, a lack of N promotes the synthesis of carbon-based secondary metabolites, such as terpenoids and phenols. Radusiene et al. [38] showed that the accumulation of catechin and chlorogenic acid in *Hypericum pruinatum* L. decreased with N application (0 ∼ 120 kg N/ha). Leser and Treutter [39] detected high flavonol concentrations in apple leaves under low N levels, which is consistent with our findings. However, Radusiene et al. [38] reported that N application (120 kg N/ha) significantly reduced the content of secondary metabolites in *Hypericum pruinatum* L. This difference in results is probably due to the differences in N application levels and species between the studies. Our results also showed that the metabolites that differentially accumulated after N fertilization were mainly flavonoids, which was consistent with previous studies [20, 37]. Wang et al. [20] showed that the flavonoid content increased in the upper leaves of tobacco plants with increasing pure N usage from 22.5 kg/hm^2^ to 67.5 kg/hm^2^. However, with increasing N fertilization beyond 75 mg N/plant, the synthesis of flavonoids decreased in *L. polystachyus*. These observations collectively suggest differences in sensitivity between plant species.

### The molecular mechanism leading to phlorizin biosynthesis in *L. Polystachyus* leaves under N application

**Fig. 8:**
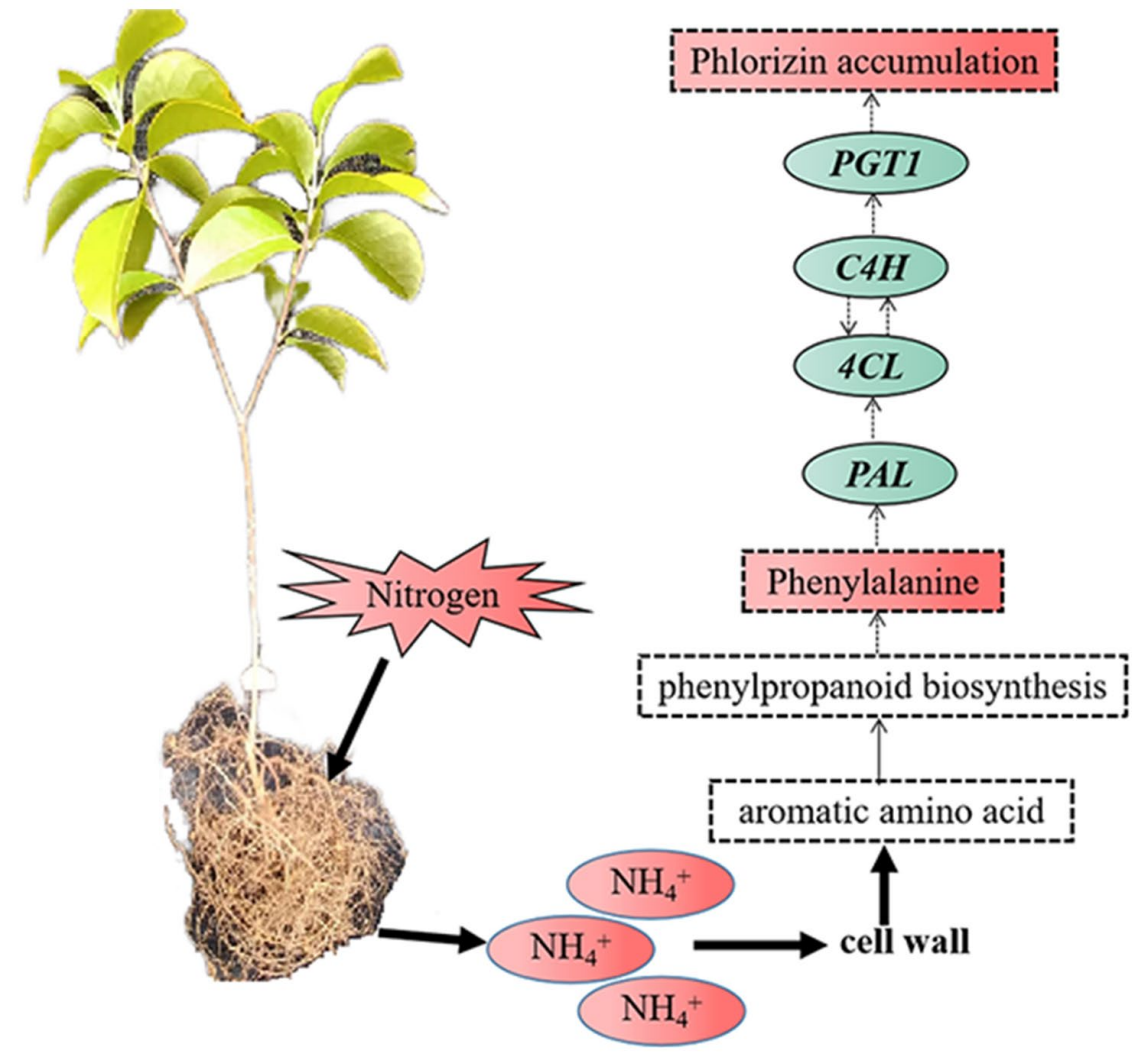
Connection between flavonoids and nitrogen metabolism in *L. polystachyus*. The solid and dashed lines represent primary and secondary metabolism, respectively

Several pharmacological studies have shown that *L. polystachyus* leaves contain various nutrients and bioactives compounds, notably flavonoids, including phlorizin and trilobatin, which constitute a significant portion of the plant’s bioactive profile [11]. Phlorizin is well known for its antibacterial, anti-inflammatory [11], antioxidant [7], hypoglycemic, hypolipidemic [40], and anticancer properties [8]. The mechanisms underlying the effects of N fertilization on flavonoids in plants have been investigated through transcriptomic, metabolomic, or multiomic methods. Studies have suggested that N application upregulates *PAL, 4CL*, and other genes and modulates flavonoid biosynthesis [41]. Our results support these findings, showing that optimal N treatment boosts the expression of flavonoid synthesis structural genes, such as *PAL* and *4CL*, which are instrumental in the early stages of phlorizin synthesis, and *PGT1*, a structural gene directly involved in phlorizin production. Therefore, we propose a hypothetical synthesis pathway (Fig. 8), where N treatment can promote the expression of these crucial genes, thereby facilitating the synthesis of phlorizin in *L. polystachyus*. In tobacco, Zhang et al. [37] reported that a low N fertilization level (60 kg/hm^2^) enhanced the expression of genes (*PAL*, *C4H*, *4CL*) involved in flavonoid metabolism in the leaves at the early stages of treatment (50 days and 83 days). Similarly, our results also revealed an increase in *PAL-* and *4CL-*encoding gene expression within 90 days of treatment at a low N level (75 mg/plant), indicating that proper N fertilization in the short term promotes *PAL* and *4CL* expression and enhances flavonoid synthesis. This observation confirms that N fertilization regulates the content of metabolites and the metabolism and synthesis of flavonoids in plants by influencing the activity of related enzymes.

The phlorizin biosynthetic pathway is an important branch of the flavonoid pathway, starting with phenylpropanoid biosynthesis [22]. Researchers have reported the effects of abiotic factors on flavonoids using transcriptomic, metabolomic, or multiomic approaches [18, 37]. For example, Hung et al. reported the accumulation of various flavonoids and high expression of associated genes (*F3H*, *FNS*, *UFGT*) in N-deficient *Camellia sinensis* L [18]. , indicating a correlation between N levels and flavonoids. However, the influence of N treatment on the flavonoid content of *L. polystachyus* was unclear. For the first time, we integrated transcriptome and metabolome data and revealed that the DEGs, including one *PAL* gene, one *C4H* gene, two *4CL* genes, one *CHI* gene, four *HCT* genes, and two *PGT1* genes, were related to phlorizin synthesis in *L. polystachyus*. These results were consistent with those of Zhang et al. [14], who identified PAL and 4CL as the key enzymes associated with phlorizin synthesis in *L. polystachyus.* Specifically, N fertilization increased the expression levels of *PAL*, *4CL*, and *PGT1* but decreased those of *C4H*, *CHI*, and *HCT*, leading to increased phlorizin synthesis. In addition, our results revealed four *PAL-*encoding genes and five *4CL*-encoding genes in *L. polystachyus* under N fertilization. We also found that the same enzyme was encoded by multiple genes, which are likely different splicing transcripts of a specific gene family [42].

## Conclusions

We used metabolomics and transcriptomics to analyze the mechanism regulating phlorizin biosynthesis in *L. polystachyus* leaves under N fertilization. The following are our major conclusions:

1. The *L. polystachyus* leaf phlorizin content first increased but then decreased with increasing N fertilization. The application of N at a 75 mg/plant rate improved plant growth and resulted in significantly greater phlorizin content than at other N application rates, indicating that 75 mg/plant N was the most appropriate application rate for *L. polystachyus*.
2. UPLC-MS/MS analysis revealed 42 significantly different flavonoids between the plants grown without and with N fertilization, including 19 upregulated and 23 downregulated flavonoids. Most importantly, N application increased the numbers of primary bioactive metabolites, especially phlorizin.
3. Integrated metabolomics and transcriptomics revealed that most DEGs under N fertilization positively regulated flavonoid biosynthesis. Genes upstream of the phlorizin biosynthetic pathway (*PAL*, *4CL*, and *PGT1*) were upregulated after N fertilization, whereas those downstream (*C4H*, *CHI*, and *HCT*) were downregulated. These results confirmed that *PAL*, *4CL*, and *PGT1* are related to phlorizin synthesis in *L. polystachyus* under N application.

## Materials and methods

### Plant materials and sampling

A pot experiment was conducted from November 2021 to April 2022 in a greenhouse maintained at a constant temperature of 20℃ at the Experimental Center of Subtropical Forestry, Chinese Academy of Forestry (27°49′ N, 114°39′ E, 104 m altitude), Xinyu city, Jiangxi Province. The plants were grown in red and yellow soil from the experimental forest farm under the mountain. Approximately 3 kg of the dried, ground, and sieved (5 mm × 5 mm mesh) soil was added to each PVC pot (15 cm × 15 cm). One-year-old local bare root seedlings of *L. polystachyus* with strong ecological adaptability obtained from Anfu County (Ji’an city) were planted in pots in November 2021 following the conventional method (one plant/pot). Water and N fertilizer were applied once every month from January to March 2022.

The experiment was established with seven treatments, including six N fertilization levels (25, 75, 125, 175, 225, and 275 mg/plant) and a control without N fertilization; 42 replicate plants were maintained per treatment. Urea (total N content ≥ 46%; Henan Jinkai Chemical Company, Kaifeng, Henan, China) was dissolved in water and applied to the plant roots through plastic pipes inserted at a soil depth of 5 cm, and fertilizer was applied once a month from January to March 2022. In April 2022, the plant ground diameter, height, and crown width were measured, and leaf and soil samples were collected. Young leaves (30 g) were collected from all 42 plants of each treatment and divided into three biological replicates. These leaves were dried and used to determine the content of water and phlorizin. Another set of three leaves was collected from each treatment, immediately frozen in liquid nitrogen, brought to the laboratory, and stored in a − 80 °C freezer for subsequent transcriptome and metabolome analyses; three such replicates were maintained per treatment in this analysis. In addition, 50 g of soil was collected from each pot to determine the total N and available N content; soil samples were collected from three pots per treatment.

The content of phlorizin in the leaf samples was determined by high-performance liquid chromatography (LC20A HPLC; Shimadzu Corporation, Japan). The expression patterns of genes were determined via RNA sequencing and verified using qRT‒PCR. The leaf samples collected were used for metabolomic analysis, with three biological replicates per treatment. The total N content of the soil was determined using the Kjelfeld method [43]. The ammonium N and nitrate N in the soil were measured using an AA3 flow analyzer, and the sum of these two variables was calculated to determine the content of available soil N [44].

### Sample processing for metabolomic analysis

*L. polystachyus* leaves were freeze-dried under vacuum (ScientZ-100 F) and crushed using a mixer mill (MM 400, Retsch) at 30 Hz for 1.5 min with zirconia beads. Approximately 50 mg of the leaf powder was incubated in 1.2 mL of 70% methanol at 4 °C for 12 h and vortexed every 30 min for 30 s (6 vortices in total). The extract was then centrifuged at 12,000 rpm for 3 min, and the supernatant was collected after allowing it to stand for 30 min. The sample was further filtered through a 0.22 μm organic filter membrane and stored in an injection vial. Finally, the sample was used to analyze the metabolites [45].

### UPLC-MS/MS analysis

The metabolome of the sample was analyzed using a UPLC-MS/MS system (SHIMADZU Nexera X2, https://www.shimadzu.com.cn/; MS/MS, Applied Biosystems 4500 OTRAP, http://www.appliedbiosystems.com.cn/) [28] equipped with an Agilent SB-C18 chromatographic column (1.8 μm, 2.1 mm × 100 mm). A mixture of formic acid in water (0.1%) and acetonitrile in 0.1% formic acid was used as mobile phases A and B, respectively, and elution was carried out using the following gradient program: 5% B for 0 min, 95% B for 9.0 min, 5% B for 10.00–11.10 min, and 5% B for 14 min. The flow rate used was 0.35 mL/min, the column temperature was 40℃, and the injection volume was 4 µL. The mass spectrometer was operated with the following parameters: Electrospray ion source (ESI) temperature, 550 °C; ion spray (IS) voltage, 5500 V (positive ion mode)/-4500 V (negative ion mode); collision-induced ionization parameters as high; ion source gas I (GSI), 50 psi; gas II (GSII), 60 psi; and curtain gas (CUR), 25 psi [28]. The instrument was debugged in triple quadrupole (QQQ) mode using 10 µmol/L polyethylene glycol and calibrated in linear ion trap (LIT) mode using 100 µmol/L polyethylene glycol. Then, the QQQ scans were acquired in MRM mode (colliding gas medium), and the clustering potential (DP) and collision energy (CE) of the MRM ion pair were optimized. A specific set of MRM ion pairs was examined based on the metabolites eluted within each period.

### Qualitative and quantitative analyses of metabolites

The metabolites in the samples were quantified using the instrument in MRM mode, as described previously [24, 28]. The self-built Metware database was used to detect and annotate the metabolites [46, 47]. As phlorizin was not in the publicly available database, a new library was built using a standard sample (No. S31392, Shanghai Yuanye Bio-Technology Co., Ltd., China), and the details were incorporated into the self-built MWDB database. Then, the secondary spectral information was used to quantify the metabolites, referring to the MWBD and other publicly available databases. The characteristic ions of each metabolite were screened by triple quadrupole, and their signal intensities (CPSs) were obtained using a detector. Any interference from nontarget ions (K^+^, Na^+^, and NH_4_^+^) was eliminated while quantifying the metabolites. The MS file of the sample was opened with Analyst 1.6.3 software, and the chromatographic peaks were integrated and adjusted. Finally, the integrated data were exported to obtain the relative content of the metabolite. Orthogonal partial least squares discriminant analysis (OPLS-DA) and principal component analysis (PCA) were performed to assess the differences between the samples.

### Differential metabolite analysis

The metabolites that differentially accumulated in the N-fertilized samples compared with the control samples were further screened based on the fold change and variable importance in the projection (VIP) values (OPLS-DA model) [48]. The built-in function in R software (www.r-project.org/; scale = True) was used to perform PCA, and the OPLSR function of the MetaboAnalystR package was used to analyze the processed chromatographic peak areas (log_2_ transformed, Mean Centering) [49]. Metabolites with fold changes ≥ 2 and ≤ 0.5, and VIP ≥ 1 were identified as differential metabolites [45]. Furthermore, these differentially accumulated substances were annotated based on the Kyoto Encyclopedia of Genes and Genomes (KEGG) database [50].

### RNA sequencing and annotation

Total RNA was isolated, and cDNA libraries were sequenced according to the methods of Wang et al. [51]. First, the leaf tissue was ground on dry ice, and RNA was extracted using TRIzol reagent (Invitrogen, CA, USA). Half of the extracted RNA was treated with DNase (Takara, Dalian, China) to remove DNA. Then, the purity of the extracted RNA was determined using a NanoPhotometer 2000 spectrophotometer (GuangZhou Sopo Biological Technology Co., Ltd., China), and the quality was assessed on an Agilent 2100 bioanalyzer (Agilent Technologies, CA, USA). Furthermore, the ribosomal RNA was removed from the total RNA to obtain the mRNA. cDNA was synthesized from the mRNA, and the adaptors were ligated to both ends using a cDNA synthesis kit (TaKaRa, Dalian, China). Finally, the different cDNA samples were pooled according to the standard sequencing by synthesis (SBS) protocol and sequenced on an Illumina HiSeq 4000 platform. The user-friendly RSEM (RNase by expectation maximization) package was used to integrate the unigenes obtained from the transcriptome database.

### Screening of DEGs

The DEGs between the control and fertilized groups were identified using the DESeq2 R package [52, 53] by importing the unstandardized read counting data of genes. After analyzing the differences, p values were adjusted for the false discovery rate (FDR) using the Benjamini‒Hochberg method. Finally, the genes with a |log2fold change| ≥ l and an FDR < 0.05 were identified as DEGs. A volcano plot was generated to display the overall distribution of DEGs between the control and fertilized samples, and scatter plots were generated to visualize the enriched KEGG pathways.

### Integrated transcriptome and metabolome analysis

To further examine the connection between gene expression and metabolite content, the DEGs and the differentially accumulated metabolites associated with the phenylpropanoid biosynthesis (ko00940) and flavonoid biosynthesis (ko00941) pathways were mapped to the KEGG database. In addition, the Pearson correlation coefficient between gene expression and metabolite content was calculated. Finally, a network was constructed using associations with a Pearson correlation coefficient (PCC) > 0.8 and *P* < 0.05 and visualized using Cytoscape software version 3.72.

### Quantitative real-time PCR

The DEGs associated with phlorizin synthesis identified in this study were verified by qRT‒PCR (ABI 7500 Real-time PCR instrument, USA) using primers synthesized by Hangzhou AiTing Biotechnology Co., Ltd. Company (Table S1). The reaction mixture consisted of 10.0 µL of 2x SYBR Premix Ex Taq, 0.8 µL of forward primer (10 µM), 0.8 µL of reverse primer (10 µM), 2.0 µL of diluted cDNA, and 6.4 µL of ddH_2_O. qRT‒PCR was carried out using the following program: 95℃ for 30 s, followed by 40 amplification cycles at 95℃ for 5 s and 60℃ for 60 s. The dissociation curve was obtained using the following program: 95℃ for 5 s and 60℃ for 1 min. Several reference genes were selected for the preliminary experiments. Finally, the relative expression levels of genes were analyzed using the 2^−ΔΔCt^ method with the reference gene UBI [54]; each sample was analyzed using three technical replicates.

### Statistical analysis

SPSS 23.0 software was used for descriptive statistical analysis, one-way analysis of variance (ANOVA), and Fisher’s least significant difference (LSD) test. The differences in the growth parameters and bioactive components of *L. polystachyus* under different N fertilization levels were statistically significant at *P* < 0.05. The metabolite data were standardized using unit variance (UV) scaling, and a heatmap was drawn using the pheatmap package in R to visualize the differences in the metabolites. Hierarchical clustering was applied to the selected DEGs, and the results were visualized using a heatmap.

### Electronic supplementary material

Below is the link to the electronic supplementary material.

Supplementary Material 1

Supplementary Material 2

Supplementary Material 3

Supplementary Material 4

Supplementary Material 5

## Data Availability

The plant materials were grown in our resource nursery. These materials are available from the corresponding author upon reasonable request. The datasets generated during the study are available in the National Genomics Data Center repository (PRJNA961833, https://www.ncbi.nlm.nih.gov/bioproject/PRJNA961833).
